# Profiling of CSF: Small Subgroups

**DOI:** 10.1371/journal.pmed.0030470

**Published:** 2006-10-31

**Authors:** Dave Hambridge

This very encouraging article [1] is based on relatively small subgroups of patients. That in itself does not invalidate the conclusions, providing that the subjects in each group have the same illness.

I recently reported that many detained psychiatric inpatients had not been fully investigated to exclude organic causes of their seeming first episode of schizophrenic-like psychosis [2].

These authors state that their patients had DSM-IV–diagnosed schizophrenia, which excludes organic causation. What investigations did they do to ensure that their clinical sample had no organic precipitants?
